# One in a million, or one in thousand: What is the morbidity of rabies in India?

**DOI:** 10.7189/jogh.02.010303

**Published:** 2012-06

**Authors:** Janie M. Baxter

**Affiliations:** Centre for Population Health Sciences, University of Edinburgh, Edinburgh, Scotland, UK

Rabies is the 10th biggest cause of death due to infectious diseases worldwide [1]. It is estimated that 2.5 billion people across 100 countries are at risk of contracting rabies [1]. The annual death toll is around 50 000–60 000, with 99% occurring in tropical developing countries [1]. Around 36% of these rabies related deaths occur in India every year with dog bites being responsible for 95-97% of these cases [2,3]. The annual estimated number of dog bites in India is 17.4 million, leading to estimated 18 000–20 000 cases of human rabies per year [4]. Rabies is a fatal condition with no cure, but there are preventive interventions to reduce its burden, although they are not well adopted in India. As a result, India has the largest contribution to worldwide rabies mortality [5-7]. Across Asia the annual expenditure due to rabies is estimated to be reaching 563 million USD [3].

Rabies typically affects the most vulnerable members of society, children and lower socio-economic classes [3]. This is likely due to poor knowledge and uptake of preventive measures. Studies have shown only around 70% of the population of India have heard of rabies, only around 30% knew to wash wounds after animal bites and a large proportion were not compliant with treatment [5]. Furthermore, rabies is not a notifiable disease in India, which makes it probable that the true burden has been underestimated [7]. Although there have been reviews focusing on rabies burden in India, the majority were published prior to 2000. They all pointed to large discrepancy between estimates of rabies burden in India, which makes it difficult for policy makers to understand the scale of the problem and plan how to tackle it. As rabies is an acute condition and its control is centered on preventive measures, incidence is the most appropriate measure of its burden in the context of improving health policy.

Over the past decade, I could only identify six studies that seemed to report the incidence of rabies in different parts of India [8-13]. I use them here to try to discuss what would be a reasonable estimate based on their reported results, but perhaps equally importantly, to expose the challenges of understanding and assessing rabies morbidity in a low-resource setting. **Table 1** shows that the case definitions used in each study were poorly reported across the board. It is also worth noting that some studies estimated the incidence of animal bites as a proxy for rabies incidence, as the latter data was not known. In order to compare estimates of rabies incidence across the 6 included studies it was necessary to first standardise all of the results. As two of the studies already reported rabies incidence as the annual number of cases per 100 000 of population, I decided to standardise all of the results to these units. All studies results were reported per year.

**Table 1:** Case definitions used in studies of rabies in India in the past decade

Study title	Case definitions	Standardized estimate of annual rabies incidence and units
Assessing the burden of human rabies in India: results of a national multi-center epidemiological survey [8].	Case definition not given. Rabies diagnosis from the records of 22 infectious diseases hospitals from all regions of the country for 1992—2002.	2 per 100 000 population
Re-evaluating the burden of rabies in Africa and Asia. [9]	Case definition not given.	130 per 100 000 population
An epidemiological study of animal bites in India: results of a WHO sponsored national multi-centric rabies survey [10]	Animal bite reported in the previous year in each household surveyed. Report based on memory recall of reliable, responsible adult or available home records.	1700 per 100 000 population
A survey of hospitals managing human rabies cases in India [11].	Case definition not given. Cases were identified from medical records across 23 medical centers.	0.05 per 100 000 population
Human Rabies in Delhi [12].	Rabies diagnosis was made on the basis of exposure history and presenting clinical features.	0.88 per 100 000 population
Epidemiology of human rabies cases in Kolkata with its application to post prophylaxis [13].	Case definition not given. Rabies cases identify at Kolkata’s single referral center.	3.48 per 100 000 population

There was an incredible variation in the standardised measure of incidence across the studies. **Table 1** shows the standardized estimates for each study, ranging from 0.05 to 1700 rabies cases per 100 000 population. If the sample size is taken into account, then the weighted mean calculated for the 6 studies was 128.74 per 100 000 , but it was mainly affected by the 2 largest estimates of rabies incidence, as these had the second and third largest sample sizes. The simple median is 2.74 per 100 000. Unsurprisingly, the 2 most extreme values were produced from the studies that estimated the incidence of animal bites as a proxy, and did not measure the incidence of human rabies directly. As a result, those two studies are likely to greatly over estimate the incidence of rabies. If those two studies are ignored, then the mean number of annual rabies cases from the remaining 4 studies is 1.6 per 100 000 and the weighted mean is 2 per 100 000.

**Figure:**
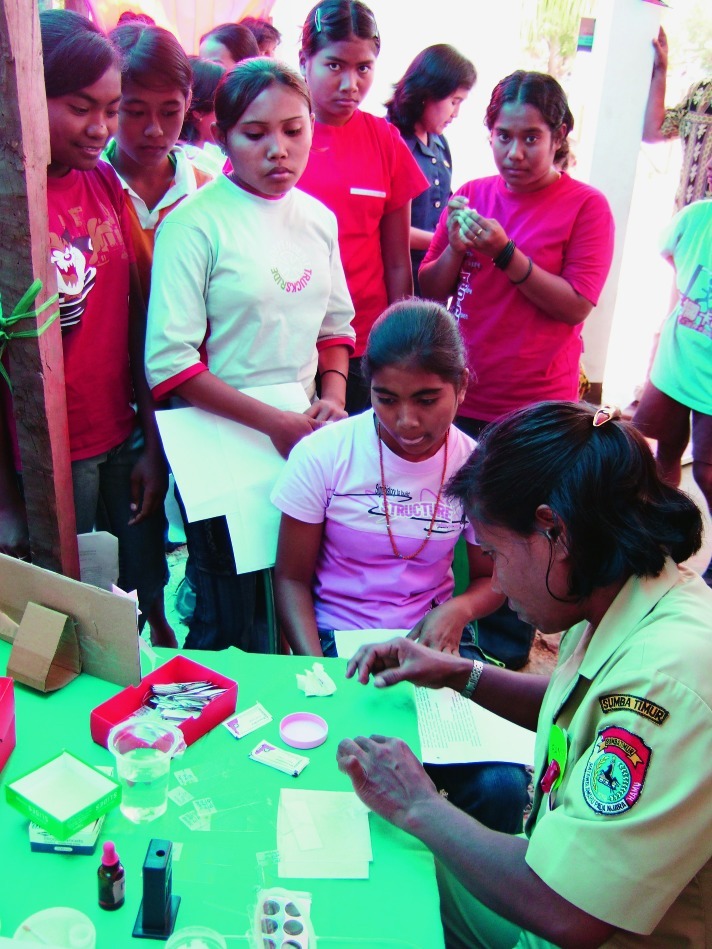
Photo: Courtesy of Dr David Hipgrave, personal collection

I propose that the median of 2.74 rabies cases per 100 000 people annually may be a fair estimate of rabies burden from the available evidence. All of the studies were retrospective cohort studies, with the exception of one prospective cohort, which is a relatively robust study design. They were all also conducted over at least a 1-year period and therefore provide a reliable estimate of annual incidence. 3 of the 6 studies contained very large study populations, including one study that contained population-wide data, leading to increased precision of the estimate. Four of the studies aimed to estimate the incidence of rabies across India and as such they used multi-centered approach across various regions. However, two of the studies focused the populations of single areas of Delhi and Kolkata, which are not likely to be demographically similar or at similar risk of rabies as the Indian population as a whole. All but one of the studies recruited participants from hospitals, this biases the study as only the incidence of those attending hospital can be calculated. This may exclude those with poorer education, access to health care or those who seek traditional healing who may also be more likely to be exposed to rabies. Across all 6 studies there was potential for error and bias in the measurement of incidence particularly as none of the studies outlined strict case definitions to ensure cases of rabies were correctly identified. Also the reporting of rabies in India is known to be poor and it is likely than many cases went unreported during the study period [7]. The influence of chance was not well addressed in any of the studies with no P-values or confidence intervals reported for any of the analyses preformed. An additional source of potential bias is that 3 of the 6 identified studies were published by the same author.

Clearly, further research will be required in order to produce a better estimate of the incidence of rabies in India. This could be facilitated by making rabies a notifiable disease in order to have population-wide data of confirmed cases [14-16]. As the disease is incurable, it is important to focus policy and planning on reducing the incidence of exposure to rabies and promoting awareness and behaviors which can help to prevent the disease. It would be necessary to consult demographics of rabies exposure and barriers to treatment to best inform these changes [17]. For example vulnerable populations should be targeted. This includes those of lower socio-economic class, living in rural areas, living in areas of high human:dog density and children [18,19]. Considering some of the barriers to treatment outlined in the introduction, education is one important way of reducing rabies incidence [5]. This could include educating people about the contraction of rabies, underlining the importance of seeking treatment, advising washing animal bites with soap and water and avoiding the application of harmful traditional remedies. There is also evidence that further education is needed among doctors [20]. Other important areas to be tackled by policy are the control and vaccination of India’s burgeoning dog population and encouraging the use of cell culture vaccines over sheep brain vaccines which has hopefully taken place due to the discontinuation of sheep brain vaccine by the government in 2004 [10,21].
